# Associations between blood essential metal mixture and serum uric acid: a cross-sectional study

**DOI:** 10.3389/fpubh.2023.1182127

**Published:** 2023-08-21

**Authors:** Dongmei Wang, Yue Li, Hualin Duan, Shuting Zhang, Lingling Liu, Yajun He, Xingying Chen, Yuqi Jiang, Qintao Ma, Genfeng Yu, Siyang Liu, Nanfang Yao, Yongqian Liang, Xu Lin, Lan Liu, Heng Wan, Jie Shen

**Affiliations:** ^1^Department of Endocrinology and Metabolism, Shunde Hospital, Southern Medical University (The First People's Hospital of Shunde), Foshan, Guangdong, China; ^2^Department of Endocrinology, Guangdong Provincial People’s Hospital (Guangdong Academy of Medical Sciences), Southern Medical University, Guangzhou, China

**Keywords:** manganese, magnesium, copper, essential metal mixture, interactions, serum uric acid

## Abstract

**Introduction:**

Although several studies have explored the associations between single essential metals and serum uric acid (SUA), the study about the essential metal mixture and the interactions of metals for hyperuricemia remains unclear.

**Methods:**

We performed a cross-sectional study to explore the association of the SUA levels with the blood essential metal mixture, including magnesium (Mg), calcium (Ca), iron (Fe), copper (Cu), zinc (Zn), manganese (Mn) in Chinese community-dwelling adults (n=1039). The multivariable linear regression, the weighted quantile sum (WQS) regression and Bayesian kernel machine regression (BKMR) were conducted to estimate the associations of blood essential metals with SUA levels and the BKMR model was also conducted to estimate the interactions of the essential metals on SUA.

**Results:**

In the multivariable linear regression, the association of blood Mg, Mn, and Cu with SUA was statistically significant, both in considering multiple metals and a single metal. In WQS regression [β=13.59 (95%CI: 5.57, 21.60)] and BKMR models, a positive association was found between the mixture of essential metals in blood and SUA. Specifically, blood Mg and Cu showed a positive association with SUA, while blood Mn showed a negative association. Additionally, no interactions between individual metals on SUA were observed.

**Discussion:**

In conclusion, further attention should be paid to the relationship between the mixture of essential metals in blood and SUA. However, more studies are needed to confirm these findings.

## Introduction

Serum uric acid (SUA) is the end-product of purine metabolism, and hyperuricemia can result from either increased production or decreased excretion of SUA (1). In recent years, the prevalence of hyperuricemia has sharply increased. Epidemiological investigations have revealed that the prevalence of hyperuricemia in America increased from 19.1% (1988–1994) to 21.5% (2007–2008) (2). A previous meta-analysis reported that the prevalence of hyperuricemia in Chinese individuals was 13.3% (3). It is widely recognized that high SUA levels are closely associated with various diseases, including gout (4), diabetes, hypertension (5), chronic kidney disease (6), non-alcoholic fatty liver disease, liver fibrosis (7), and adverse cardiovascular outcomes (8). Therefore, it is crucial to identify the risk factors for hyperuricemia.

In recent years, there has been an increasing amount of epidemiological research investigating the potential relationship between essential metals and hyperuricemia. A cross-sectional study on middle-aged Chinese indicated that dietary zinc (Zn) intake was correlated with hyperuricemia negatively (9). Another cross-sectional study suggested the positive relationship between copper (Cu) and hyperuricemia in middle-aged and older adults population (10). One recent study found magnesium (Mg) inversely correlated with the risk of new-onset hyperuricemia (11). However, these studies only assessed the individual association between hyperuricemia and essential metals, rather than evaluating the mixture of the metals and the possible interactions between them.

In China, an increasing number of people are taking multivitamin-mineral tablets for wellness without measuring the concentrations of the metals in the blood (12). People are always exposed to multiple metals daily, possibly interacting with each other. Previous studies only focused on the associations between co-exposure to heavy metals (lead, cadmium, mercury) and SUA (13–15). A recent study found the potential interactions between manganese (Mn) and cobalt (Co), Mn and chromium (Cr), Mn and Zn, and Cr and Co on hyperuricemia (9, 16). However, the associations between blood essential metal mixture and SUA have yet to be studied so far, as well as the interactions of the essential metals.

In the current study, we chose a metal mixture including Mg, calcium (Ca), iron (Fe), Cu, Zn, and Mn, which are commonly tested clinically. We aimed to explore the associations between SUA and the blood essential metal mixture and the potential interactions of the essential metals, including Mg, Ca, Fe, Cu, Zn, and Mn, on SUA levels in Chinese community-dwelling adults.

## Materials and methods

### Study population

This cross-sectional study was conducted at www.chictr.org.cn (ChiCTR2100054130) using data from participants who were enrolled in 2021 from Lecong, Shunde District, Foshan, China, using a stratified cluster sampling method (17, 18). Initially, 1,111 potential participants aged ≥18, who had lived in Shunde for more than 6 months and were not pregnant, were identified. Four individuals who had not undergone blood examination and 68 individuals who had taken SUA lowering drugs were excluded, resulting in a final sample of 1,039 participants (Supplementary Figure S1). The study protocol (20211103) was approved by the Ethics Committee of Shunde Hospital of Southern Medical University and was conducted in accordance with the ethical guidelines of the 1975 Declaration of Helsinki. Prior to participating in the study, written informed consent was obtained from all participants.

### Measurements and definitions

#### Data collection

A team of well-versed researchers conducted a standardized survey to gather data, encompassing demographics, lifestyle, medical history, and medication usage (19). Anthropometric characteristics, such as weight, height, and blood pressure, were measured in accordance with established protocols. Body Mass Index (BMI) was computed as weight in kilograms divided by height in meters squared (kg/m^2^). Blood pressure readings were obtained twice using an automated electronic device (HEM-752 FUZZY, Omron, China), and the mean of these measurements was selected (20).

#### Laboratory assays

All samples were collected from participants who had fasted for at least 10 h overnight between 08:00 am and 10:00 am. The samples were transported under cold chain management to a central laboratory certified by the College of American Pathologists and were centrifuged within 4 h. The SUA concentration was detected using a Beckman Coulter AU 5800 (Beckman Coulter Inc., Brea, CA, United States). The blood concentrations of six metals, including Mg, Ca, Fe, Cu, Zn, and Mn, were measured using inductively coupled plasma mass spectrometry (ICP-MS) (Thermo Fisher Scientific, iCAP RQ, Waltham, United States). Fasting plasma glucose (FPG) was assessed using a Hitachi LABOSPECT 008AS (Tokyo, JAPAN), and 2 h postprandial plasma glucose (PPG) was measured using a Hitachi 7,600 automatic biochemical analyzer (Hitachi, Tokyo, Japan). Glycated hemoglobin (HbA1c) was assessed using high-performance liquid chromatography (TOSOH, HLC-723 G8, Tokyo, JAPAN). Additionally, the serum creatinine (Scr) and blood lipid profiles, including total cholesterol (TC), triglyceride (TG), high-density lipoprotein (HDL), and low-density lipoprotein (LDL), were measured using a BS800 (Mindray, Shenzhen, China).

#### Covariates

All the models were adjusted for categorical covariates: age categories (≤40, 40–60 and > 60 years), sex (men, women), educational levels(<high school, high school, and > high school), smoking status (never, ever, and current) (21), alcohol abuse (yes, no), BMI categories (<24, 24–28, ≥28) (22), diabetes (yes, no), hypertension (yes, no), dyslipidemia (yes, no) and eGFR (<60, 60–89, ≥90). It was considered alcohol abuse if it was>30 g/day for men and > 20 g/day for women (23). Diabetes was defined as FPG level ≥ 7.0 mmol/L, PPG ≥11.1 mmol/L, or HbA1c ≥6.5% and/or self-reported history of disease diagnosis (24). Hypertension was defined if systolic blood pressure ≥ 140 mmHg or diastolic blood pressure ≥ 90 mmHg, and/or self-reported history of disease diagnosis (25). Dyslipidemia was defined as a higher level of TC (≥6.22 mmol/L), TG (≥2.26 mmol/L), LDL-C (≥4.14 mmol/L), or HDL-C (<1.04 mmol/L) and/or previous diagnosis of dyslipidemia (26). The estimated glomerular filtration rate (eGFR) was calculated using the modified 4-variable Modification of Diet in Renal Disease (MDRD) equation:


$$\begin{array}{c} \mathrm{eGFR}\left(\frac{\mathrm{ml}}{\min}/m^{2}\right)=175\times {\left(\mathrm{Scr}\right)}^{-1.154}\times {\mathrm{age}}^{-0.203}\\ \times \;\;0.742\left(\mathrm{if female}\right)\times \;\;1.212\left(\mathrm{if black}\right), \end{array}$$

where Scr was expressed as mg/dl and age was expressed in years (27).

### Statistical analysis

The data were analyzed using IBM SPSS Statistics (version 24) and R (version 4.2.2). Continuous variables were summarized using mean and standard deviations or median and interquartile range (IQR) whether the variable distribution was symmetric or not, respectively.

The Spearman correlation method was applied to assess the correlation among the different essential metal elements. To achieve normal distribution, the levels of studied metal elements were ln-transformed. The association between blood essential metals and SUA was evaluated using multivariable linear regression, weighted quantile sum (WQS) regression, and Bayesian kernel machine regression (BKMR) models. The statistical level of significance was set at 0.05 and all tests were two-tailed.

The multivariable linear regression: We performed the multivariable linear regression analysis to examine the relationship between blood essential metals and SUA. Corrected β regression coefficients and 95% confidence intervals (CIs) were calculated. Initially, a multivariable linear regression model was fitted for each essential metal, controlling for other metals and potential confounders such as age, sex, education, smoking status, alcohol abuse, BMI, diabetes, hypertension, dyslipidemia, and eGFR. We observed no significant collinearity among these variables (all VIF < 3) (Supplementary Table S1). Finally, we investigated the associations between each essential metal and SUA after adjusting for potential confounders.

BKMR model: We applied the nonparametric BKMR method to estimate the overall, single-exposure, and interactive effects of each metal element with SUA, while considering possible interactions and nonlinear associations (28). Specifically, for a continuous outcome, the model can be expressed as follows:


$$Y_{i}=h\left(z_{i1},\;\ldots \;\;\ldots ,\;z_{iM}\right)+x_{i}\beta +\epsilon_{i}$$

Where $Y_{i}\;$ represents the response for individual *i* (*i* = 1,…, n), $z_{iM}$ is the *m*th exposed variable, *h*() is the unknown exposure-response function, β is the effect of the covariates $x_{i}$ , and the residuals $\epsilon_{i}\sim N(0,\sigma^{2})$ are assumed to be independent and identically normally distributed with a common variance (29). The posterior distributions for variables were provided using 50,000 iterations of the Markov Chain Monte Carlo (MCMC) (30–33). We utilized the R package BKMR for this analysis.

WQS regression model: WQS is an approach that can evaluate the mixed effect of metals and estimate the weight of individual metals (34). The study participants were randomly allocated to a training dataset (40%, *n* = 416) and a validation dataset (60%, *n* = 623) (35). We used 1,000 bootstrap samples (*B* = 1,000) from the training dataset to calculate the weights that maximize the likelihood for the model in this analysis (36, 37). The fitted model of WQS can be expressed as follows:


$$g\left(\mu \right)=\beta_{0}+\beta_{1}\overset{c}{\underset{i=1}{\sum }}w_{i}q_{i}+\varphi z^{\prime }$$

$$\mathrm{WQS}=\overset{c}{\underset{i=1}{\sum }}\bar{w_{i}}q_{i}$$

where $\bar{w_{i}}=\frac{1}{B}\sum_{b=1}^{B}w_{i\left(b\right)}f\left({\hat{\beta }}_{1\left(b\right)}\right)$ and $f\left({\hat{\beta }}_{1\left(b\right)}\right)$ is a pre-specified “signal function” of the estimated slope parameter associated with WQS from the *b*th bootstrap sample to measure the signal strength (30, 34, 38). In the above equation, the term $\sum_{i=1}^{c}w_{i}q_{i}$ represents the overall weighted index for the set of 6 studied metals. In the second equation above, the *c* = 6 essential metal concentrations were categorized into quartiles (*qi*) and assigned a score of 0, 1, 2, or 3 for *i* = 1 to *c*. The weight for the *i*th chemical component *qi* is represented by *wi*. In the first equation, *z* refers to the covariate vector determined before the weights were estimated, and φ represents the coefficients for the covariates in *z* (34, 39). For this analysis, metals with estimated weights greater than 0.167 (1/6) were considered significant contributors to the WQS score (40). To improve the robustness of the regression parameters, we apply the repeated holdout extension of WQS with 100 repetitions. That is, we performed 100 random partitions on the dataset and repeated WQS regressions in each partition. Based on the simulated distribution, the β coefficient of the WQS index was averaged as the final estimate (41–43). The gWQS package in R was employed for this purpose.

## Results

### General characteristics of participants

Table 1 presents an overview of the participants’ general characteristics, which includes age, sex, SUA, FPG, HbA1c, education level, BMI, smoking status, alcohol abuse, hypertension, dyslipidemia, diabetes, eGFR categories, and the investigated metallic elements. Of the 1,039 participants, 438 (42.2%) were male and 601 (57.8%) were female, with a mean age of 49.18 ± 14.27 years. The average SUA level was 378.42 ± 96.80 μmol/L. In terms of BMI, the prevalence of obesity was 13.1%. 98.3% participants reported no alcohol abuse, and 81.1% had never smoked cigarettes. Among all participants, 68.0% had normal eGFR (≥90 mL/min/m^2^). Furthermore, the blood Mn, Ca, Cu and Zn were slightly left skewed, while the blood Mg and Fe were slightly right skewed.

**Table 1:** General characteristics of participants in the study.

	Total (*n* = 1,039)
Age, years	49.18 ± 14.27
Male, %	42.2
SUA, μmol/L	378.42 ± 96.80
FPG, mmol/L	5.03 (4.70,5.41)
HbA1c, %	5.70 (5.40,6.00)
Education, %	
< High school	28.8
High school	34.1
> High school	37.2
BMI, %	
Normal weight	55.0
Overweight	32.0
Obese	13.1
Smoking status, %	
No	81.1
Ever	4.7
Current	14.1
Alcohol abuse, %	1.7
Hypertension, %	33.8
Dyslipidemia, %	42.3
Diabetes, %	11.4
eGFR, %	
<60 mL/min/m^2^	2.5
60–90 mL/min/m^2^	29.5
≥90 mL/min/m^2^	68.0
Mg, mg/L	41.80 (38.60,45.00)
Mn, μg/L	12.60 (10.30,15.20)
Ca, mg/L	62.80 (58.80,67.00)
Fe, mg/L	506.00 (468.50,543.90)
Cu, μg/L	875.40 (799.20,963.60)
Zn, mg/L	6.30 (5.70,6.90)

The general characteristics of participants were summarized as mean ± standard deviation or median and interquartile range (IQR) for continuous variables and frequencies for categorical variables (%). SUA, serum uric acid; BMI, body mass index; eGFR, estimated glomerular filtration rate; FPG, fasting plasma glucose; HbA1c, glycated hemoglobin; Mg, magnesium; Mn, manganese; Ca, calcium; Fe, iron; Cu, copper; Zn, zinc.

### The associations between blood essential metal and SUA using the multivariable linear regression

**Figure 1:**
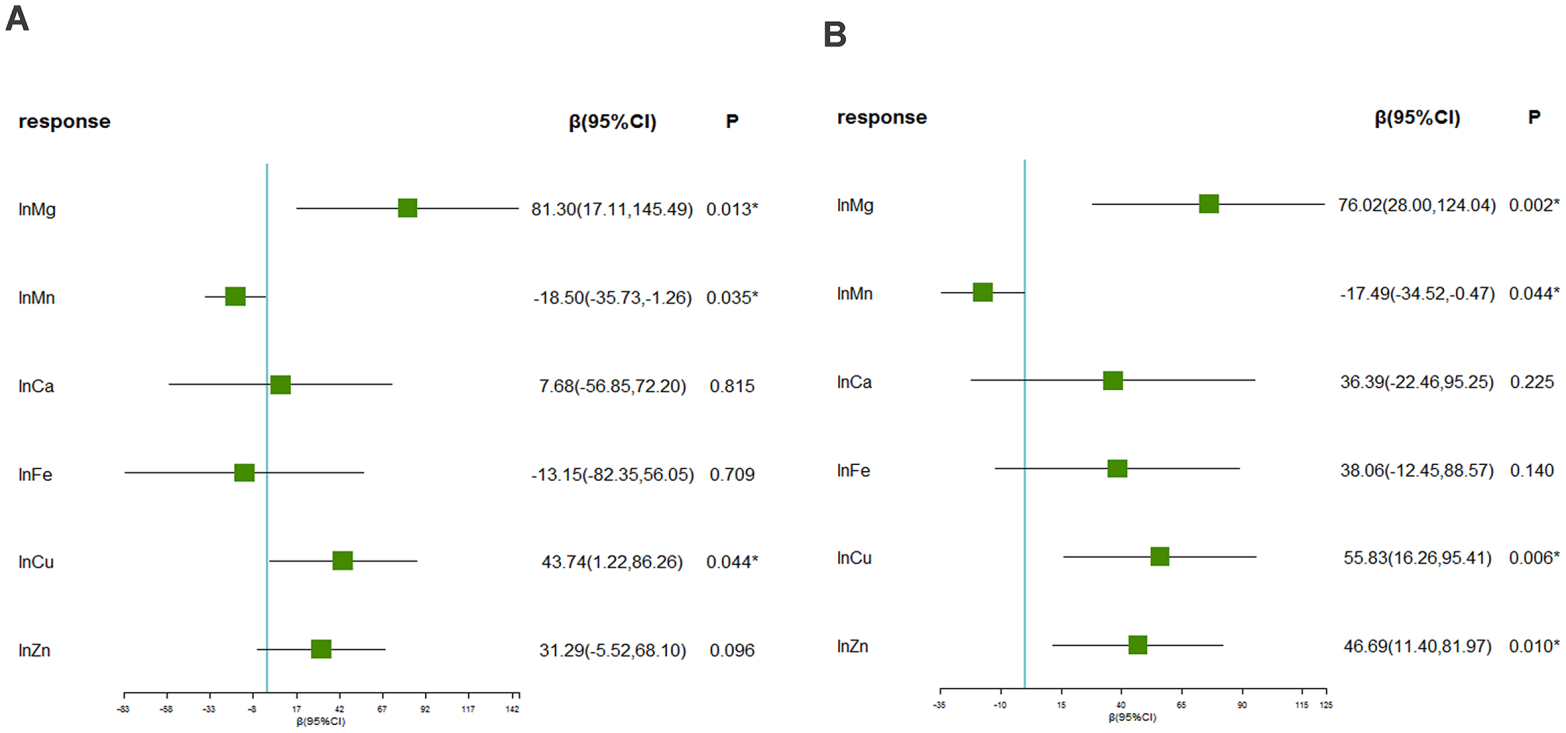
The associations between blood essential metals and SUA using the multivariable linear regression. **(A)** Linear regression considering multiple metals **(B)** linear regression considering a single metal. The levels of studied metal elements were ln-transformed to improve normal distribution. The model was adjusted for age, sex, educational levels, smoking status, alcohol abuse, BMI categories, diabetes, hypertension, dyslipidemia, and eGFR. BMI, body mass index; eGFR, estimated glomerular filtration rate; Mg, magnesium; Mn, manganese; Ca, calcium; Fe, iron; Cu, copper; Zn, zinc.

Supplementary Figure S2 displays the Spearman correlation coefficients among essential metals, which suggested the correlations ranging from weak to moderate (r: 0.01 to 0.65) (44). Figure 1 presents that the associations of blood Mg [β = 81.30 (95%CI: 17.11, 145.49), *p* = 0.013], Mn [β = −18.50 (95%CI: −35.73, −1.26), *p* = 0.035], and Cu [β = 43.74 (95%CI: 1.22, 86.26), *p* = 0.044] with SUA was statistically significant, considering multiple metals. Furthermore, the associations of blood Mg [β = 76.02 (95%CI: 28.00, 124.04), *p* = 0.002], Mn [β = −17.49 (95%CI: −34.52, −0.47), *p* = 0.044], Cu [β = 55.83 (95%CI: 16.26, 95.41), *p* = 0.006], and Zn [β = 46.69 (95%CI: 11.40, 81.97), *p* = 0.010] with SUA were statistically significant, considering a single metal.

### The associations between blood essential metal mixture and SUA using the BKMR model

**Figure 2:**
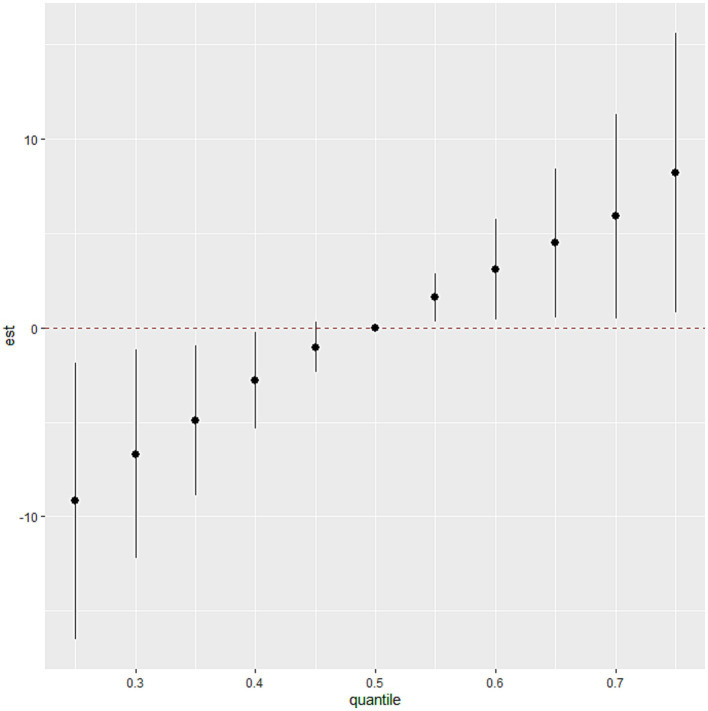
The association between the blood essential metal mixture and SUA using the BKMR model. The levels of studied metal elements were ln-transformed to improve normal distribution. The model was adjusted for age, sex, educational levels, smoking status, alcohol abuse, BMI categories, diabetes, hypertension, dyslipidemia, and eGFR. We considered the results statistically significant if the 95% confidence interval (CI) of the estimate does not include 0. BMI, body mass index; eGFR, estimated glomerular filtration rate; Mg, magnesium; Mn, manganese; Ca, calcium; Fe, iron; Cu, copper; Zn, zinc.

**Figure 3:**
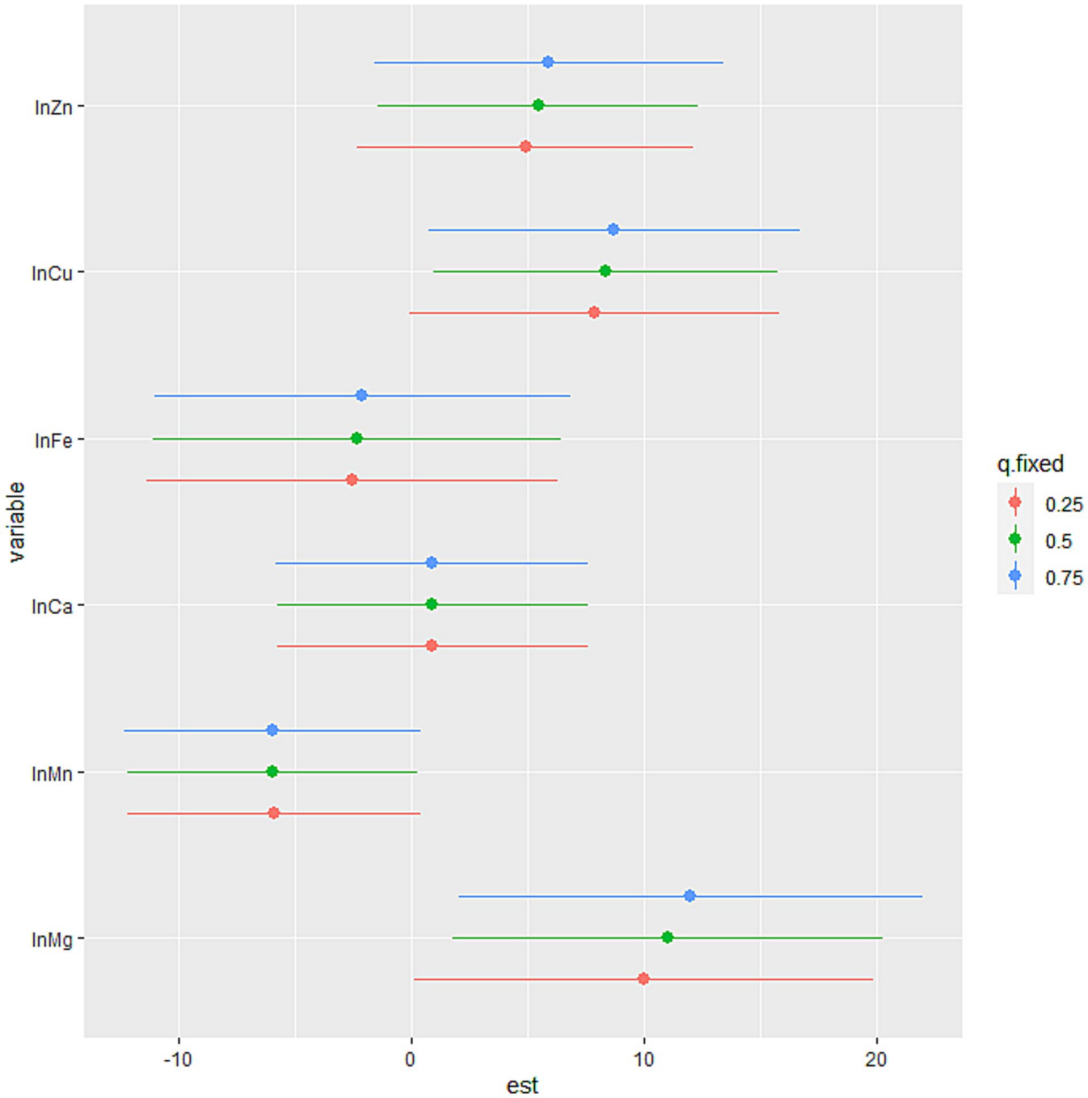
The association between the individual blood essential metal and SUA using the BKMR model. The levels of studied metal elements were ln-transformed to improve normal distribution. The model was adjusted for age, sex, educational levels, smoking status, alcohol abuse, BMI categories, diabetes, hypertension, dyslipidemia, and eGFR. The 75th percentile of the individual metal was compared to its 25th percentile, with the other essential metals at their 25th, 50th, or 75th percentile. We considered the results statistically significant if the 95% confidence interval (CI) of the estimate does not include 0. And the position of the individual metal suggested the positive or negative effect on the outcome. BMI, body mass index; eGFR, estimated glomerular filtration rate; Mg, magnesium; Mn, manganese; Ca, calcium; Fe, iron; Cu, copper; Zn, zinc.

The BKMR model revealed that higher levels of SUA were associated with increasing levels of common blood metal elements when the concentrations of blood essential metals were above the 55th percentile (Figure 2). To examine the contribution of individual exposures to the overall effect, we fixed the other elements at their 25th, 50th, or 75th percentile and identified significant relationships between Mg, Mn, and Cu with SUA. Our findings indicated that increased exposure to Mg and Cu was associated with higher levels of SUA, while increased exposure to Mn was associated with lower levels of SUA (Figure 3). Additionally, Supplementary Figure S3 illustrates no interactions between individual metals on SUA.

### The associations between blood essential metal mixture and SUA using the WQS model

**Figure 4:**
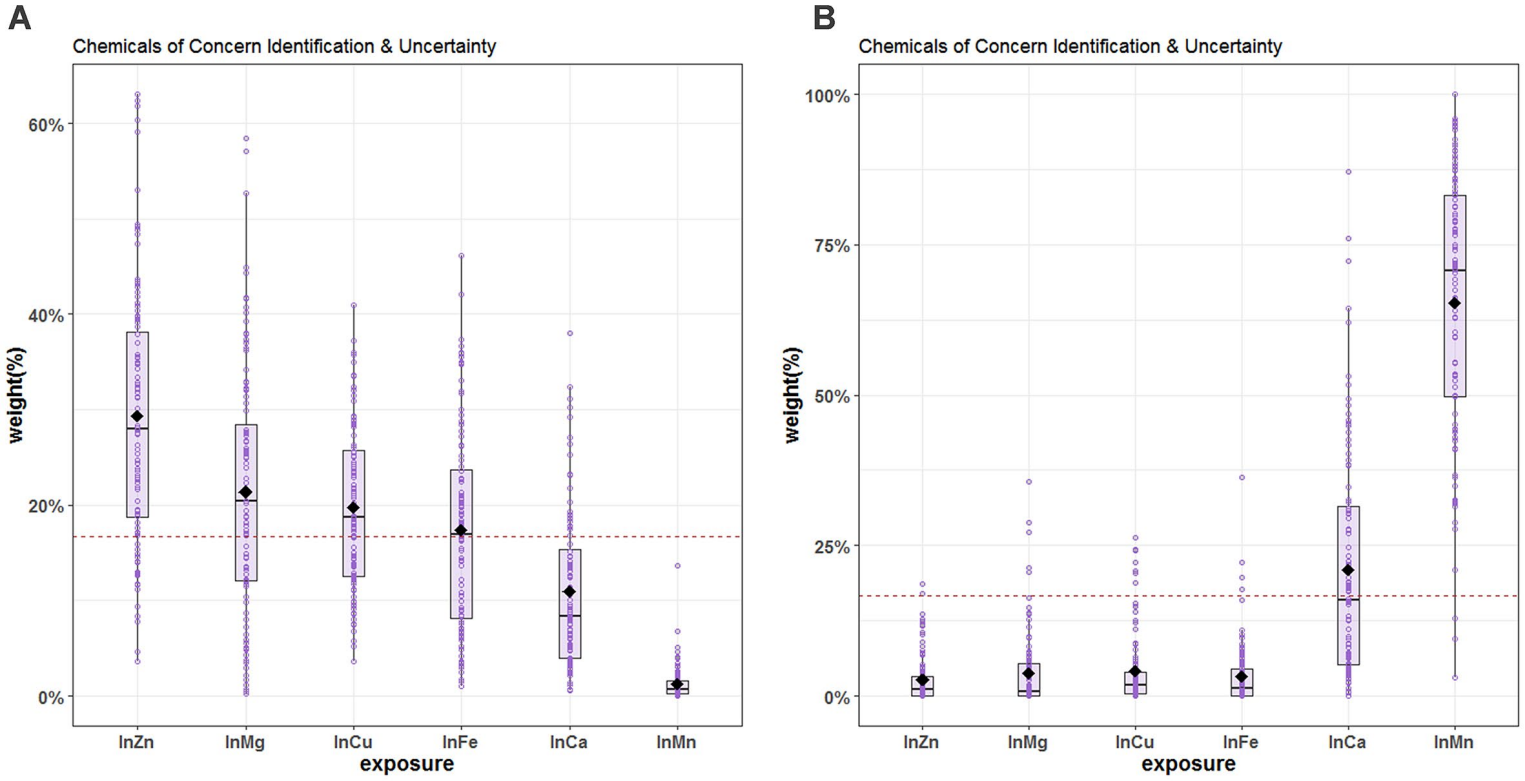
Chemical of concern identification & uncertainty for blood essential metals and SUA. **(A)** The positive direction **(B)** the negative direction. The levels of studied metal elements were ln-transformed to improve normal distribution. The hollow points represent the weight value of the 100 repetitions, and the box plots show the 25th, 50th, and 75th percentiles, and the diamonds represent the mean weights of 100 repetitions. The horizontal line represents the threshold value of the weight, and metals with estimated weights greater than threshold 1/6 (16.7%) were considered significant contributors to the WQS score. The model was adjusted for age, sex, educational levels, smoking status, alcohol abuse, BMI categories, diabetes, hypertension, dyslipidemia, and eGFR. BMI, body mass index; eGFR, estimated glomerular filtration rate; Mg, magnesium; Mn, manganese; Ca, calcium; Fe, iron; Cu, copper; Zn, zinc.

Since there is no evidence of non-linear relationship between the exposures and the outcome and that there is no interaction among the elements in the mixture, we applied the WQS regression. The WQS model demonstrated the association between the mixture of essential metals and SUA. The WQS index showed a positive and significant association using repeated holdout validation [β = 13.59 (95%CI: 5.57, 21.60)] (Table 2), indicating that the combined effect of mixed metal elements was positively linked to SUA (42). Table 3 shows the mean weights obtained by the repeated holdout. Specifically, blood Zn (29.3%), Mg (21.4%), Cu (19.7%) and Fe (17.4%) made significant contributions in positive direction. Conversely, blood Mn (65.3%) had the highest weight in the negative direction, and blood Ca (20.9%) also made a considerable contribution. What’s more, Figure 4 displays the distribution of all metal weights obtained with 100 repetitions.

**Table 2:** WQS index β coefficients and CIs by validation technique & estimation type.

Validation technique	Estimation type	β coefficient	Lower limit	Upper limit
None: train/test full dataset	Mean & SE-based 95% CI	20.30	9.50	31.10
Repeated holdout	Mean & SD-based 95% CI	13.59	5.57	21.60

**Table 3:** The mean weights obtained by the repeated holdout.

	Positive	Negative
lnZn	0.293	0.027
lnMg	0.214	0.038
lnCu	0.197	0.041
lnFe	0.174	0.032
lnCa	0.109	0.209
lnMn	0.012	0.653

Mg, magnesium; Mn, manganese; Ca, calcium; Fe, iron; Cu, copper; Zn, zinc.

## Discussion

The current study examined the associations between blood essential metals, including Mg, Ca, Fe, Cu, Zn, and Mn, and SUA levels in Chinese community-dwelling adults using three statistical approaches. In reality, different models serve different purposes, and each approach may have its own advantages and disadvantages, leading to distinct outcomes. The multivariable linear regression is a commonly used and straightforward method for analysis and interpretation of data. And the current study demonstrated a statistically significant association between blood Mg, Mn, and Cu with SUA when considering multiple metals. However, we conducted multivariable linear regression and found a positive relationship between blood Zn and SUA in the analysis of single metal. The moderate correlation between some metals might lead to result distortion (45). Also, it is not enough when considering mixed exposures and their complex interactions in a simple linear regression model (34, 45). The BKMR model, on one hand, can estimate both the interactive effects of exposures and reveal the nonlinear relationships between exposures and outcomes (30, 46, 47). In our analysis, blood Mg and Cu were positively associated with SUA, while blood Mn showed a negative relationship. WQS regression, on the other hand, is capable of assessing the overall effect and contribution of each exposure by estimating the weight of individual metals (48, 49). For instance, the combined effect of mixed metal elements was positively linked to SUA [β = 13.59 (95%CI: 5.57, 21.60)]. However, it assumes that all exposures have the same direction of associations with the outcome, which requires making assumptions in two different directions (48, 50, 51). In the current study, we found that the blood Zn (29.3%) had the highest mean weight in positive direction while the blood Mn (65.3%) had the highest mean weight in the negative direction. Overall, the results of both models were consistent and reliable, suggesting that essential metal supplementation should not be blindly undertaken. These findings provide a possible approach to preventing hyperuricemia.

The relationship between Zn and uric acid (UA) has been controversial in previous research. A study from NHANES (2001–2014, *n* = 37,215) suggested that dietary Zn intake (median: 11.82 mg/d for men; 8.45 mg/d for women) is inversely associated with hyperuricemia using 24 h dietary recall method (52). Another cross-sectional study in China (*n* = 5,168) also indicated that dietary Zn intake (median: 20.54 mg/d for men; 17.20 mg/d for women) was inversely associated with hyperuricemia in men using the food frequency questionnaire (FFQ) (9). Additionally, an animal study inferred that Zn can alleviate hyperuricemia by decreasing the activities of adenosine deaminase and xanthine oxidase (XO), and promoting UA excretion by altering intestinal flora composition (53). However, these studies had focused the dietary Zn intake instead of the blood Zn. A cross-sectional study in China (*n* = 1,046) shown that higher plasma levels of Zn might increase hyperuricemia risk (54). Another study (*n* = 6,508) also found that the urinary levels of Zn positively associated with hyperuricemia in China (55). The current study indicated a positive association between blood Zn and SUA. Our bodies have a capacity to maintain Zn homeostasis while dietary Zn intake varies (56). So the change of dietary Zn intake would not consistent with other biological samples (blood or urinary). Additionally, the positive associations between Zn and UA had been found in plasma, urinary and blood. And more studies on mechanisms were needed.

There are some researches have explored the relationship between blood Mn and SUA. One study (*n* = 3,926) reported negative associations between Mn and SUA concentration (57), consistent with our findings. Conversely, another study based on NHANES from 2011 to 2018 (*n* = 14,871) indicated that the association between blood Mn and SUA was not statistically significant (15). In accordance of a previous study, people with long-term and low-level of occupational Mn exposure showed a lower level of UA, but high-level of Mn exposure would cause nerve damage (58) which performed as parkinsonian symptoms (59). More and more studies had found that UA has a protective effect on Parkinson’s disease (PD) (59–61), and the PD case had the lower levels of UA (62). Above all, there is a hypothesis that excessive Mn exposure leads to nerve damage that ultimately reduce the levels of UA, but further research is needed to find the mechanism between UA, Mn and the nervous system.

The current study has identified a positive association between blood Cu and SUA levels. In a cross-sectional analysis of Italian adults (*n* = 1,197), it was observed that dietary Cu intake was inversely correlated with SUA levels, while serum Cu levels did not demonstrate statistical significance in relation to SUA (63). This finding is consistent with a previous cross-sectional study conducted in Chinese adults (*n* = 6,212), which suggested a positive relationship between serum Cu levels and hyperuricemia prevalence (10). The inconsistency in results may be attributed to differences in Cu exposure. The physiological mechanisms underlying the association between Cu and SUA remain to be established. Adenosine monophosphate (AMP) is converted to purine base hypoxanthine by deaminase, nucleotidase and purine nucleoside phosphorylase (PNP). Hypoxanthine is then oxidized by XO to form xanthine, which is again oxidized by XO to form the final product, UA (64). A vitro study has proposed that Cu can either inhibit or activate XO, depending on its concentration. Notably, a sharp inhibition of XO activity was observed when the Cu concentration reached 0.7 mM (65). It is plausible that different populations may have varying levels of Cu exposure, resulting in differing outcomes.

A previous study suggested an inverse relation of plasma Mg with the risk of new-onset hyperuricemia (11). Another study from American adults in 2001–2014 (*n* = 26,796) indicated that Mg intake could prevent hyperuricemia (66). However, a meta-analysis showed that exposure to Mg was not associated with hyperuricemia risk (67). The results of these studies were inconsistent with our current study, which found that the relationship of Mg with SUA was positive. The previous studies suggested that Mg is positively associated with all lipoprotein species due to the affinity of specific phospholipids head groups as a divalent cation (68). A cohort investigation demonstrated a positive relationship between hyperuricemia and dyslipidemia in the older adults population (69). Furthermore, the administration of statin therapy resulted in a significant reduction in SUA levels (70). The observed positive association between Mg and SUA may be attributed to the reported positive relationship between Mg and TG. Additionally, the discrepancy in measurement methods between our study (blood Mg) and previous studies (serum Mg) may be a potential contributing factor.

Despite the various models utilized and comprehensive adoption of results in the current study, there remain several limitations that should be noted. Firstly, given the cross-sectional design of the study, only association analysis could be performed, precluding any causal inferences. Secondly, while our sample size possessed sufficient power, the number of participants was relatively small. Lastly, it is important to note that metal ions in different valence states have varying functions, and further analyses would benefit from the detection of valence states.

In conclusion, our findings show a positive relationship between blood levels of essential metals and SUA. Specifically, we observed positive associations between SUA and blood Mg and Cu, while a negative association was found between SUA and blood Mn. Additionally, no interactions between individual metals on SUA were observed. These results highlight the need to further investigate the relationship between blood essential metals and SUA. Nevertheless, further studies are warranted to confirm our conclusions.

## Data availability statement

The raw data supporting the conclusions of this article will be made available by the authors, without undue reservation.

## Ethics statement

The studies involving humans were approved by the Ethics Committee of Shunde Hospital of Southern Medical University. The studies were conducted in accordance with the local legislation and institutional requirements. The participants provided their written informed consent to participate in this study.

## Author contributions

DW and HD conducted the data analysis. LiL, YH, XC, YJ, QM, GY, SL, NY, YoL, XL, and LaL conducted the data acquisition. DW drafted the manuscript. YuL, SZ, HW, and JS revised the manuscript. JS and HW performed the conceptualization. All authors contributed to the article and approved the submitted version.

## Funding

This work was supported by Guangdong Medical Sciences Research Foundation (A2020030); and Guangdong Basic and Applied Basic Research Foundation (2021A1515110682).

## Conflict of interest

The authors declare that the research was conducted in the absence of any commercial or financial relationships that could be construed as a potential conflict of interest.

## Publisher’s note

All claims expressed in this article are solely those of the authors and do not necessarily represent those of their affiliated organizations, or those of the publisher, the editors and the reviewers. Any product that may be evaluated in this article, or claim that may be made by its manufacturer, is not guaranteed or endorsed by the publisher.
