# Supplementary material for: Associations between blood essential metal mixture and serum uric acid: a cross-sectional study
Source: Front Public Health. 2023 Aug 21;11:1182127. doi: 10.3389/fpubh.2023.1182127 (PMC10476669; doi:10.3389/fpubh.2023.1182127)
Supplement: Supplementary file 1 [file Table_1.DOCX]

**Supplementary table 1.** The collinearity diagnosis of the covariates

| Characteristic | VIF |
| --- | --- |
| Age categories | 1.738 |
| Sex | 2.212 |
| Education level | 1.476 |
| BMI categories | 1.162 |
| Smoking status | 1.441 |
| Abused drink | 1.051 |
| Hypertension | 1.203 |
| Diabetes  Dyslipidemia  eGFR categories | 1.115  1.146  1.177 |
| lnMg | 2.066 |
| lnMn | 1.103 |
| lnCa | 1.451 |
| lnFe | 2.707 |
| lnCu | 1.417 |
| lnZn | 1.257 |

Data are presented as variance infation factor (VIF).
