# Supplementary figures and images for: Associations between blood essential metal mixture and serum uric acid: a cross-sectional study
Source: Front Public Health. 2023 Aug 21;11:1182127. doi: 10.3389/fpubh.2023.1182127 (PMC10476669; doi:10.3389/fpubh.2023.1182127)

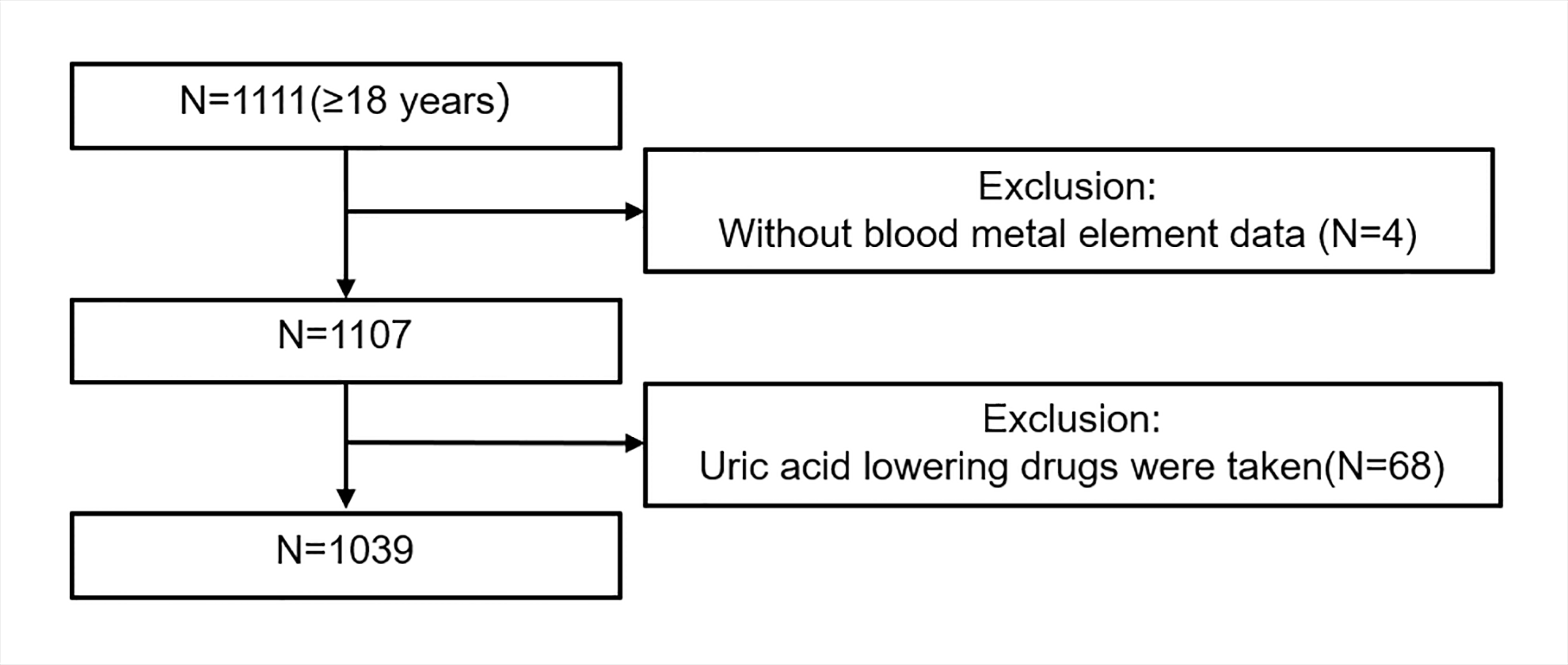

Supplement: Supplementary file 2 [file Image_1.TIF]

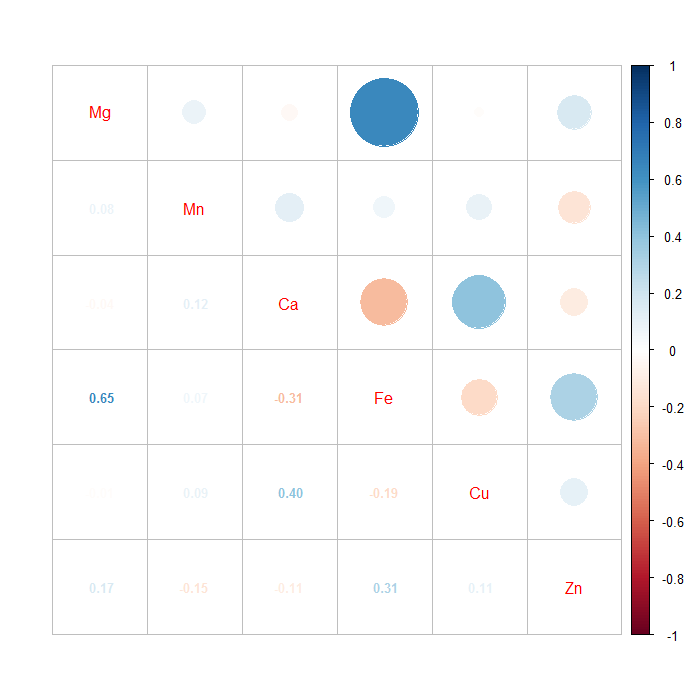

Supplement: Supplementary file 3 [file Image_2.TIFF]

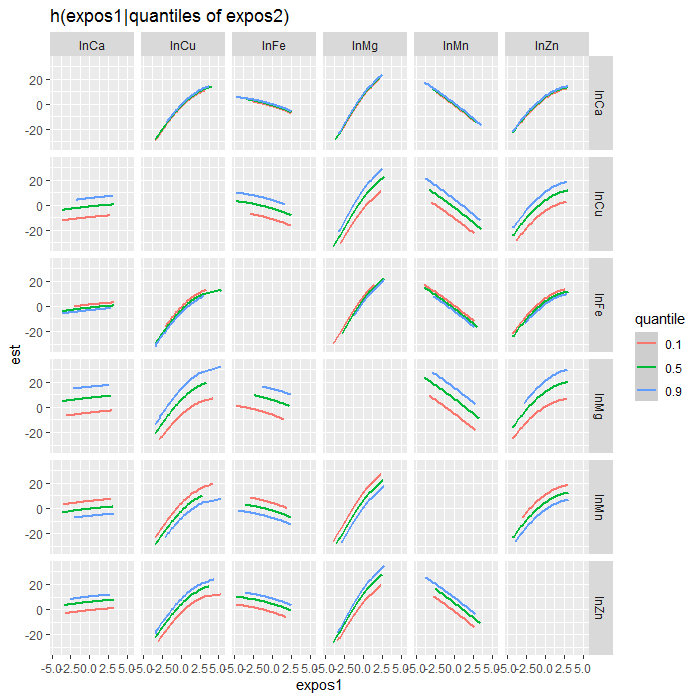

Supplement: Supplementary file 4 [file Image_3.TIFF]
